# Hyperinsulinism in a patient with a Zellweger Spectrum Disorder and a 16p11.2 deletion syndrome

**DOI:** 10.1016/j.ymgmr.2020.100590

**Published:** 2020-04-28

**Authors:** Eva M.M. Hoytema van Konijnenburg, Ilse K. Luirink, Sebastian E.E. Schagen, Marc Engelen, Kevin Berendse, Bwee Tien Poll-The, Malika Chegary

**Affiliations:** a Department of Pediatrics, Emma Children's Hospital, Amsterdam UMC, Meibergdreef 9 1105, AZ, Amsterdam, the Netherlands; b Department of Pediatrics, OLVG Hospital, Jan Tooropstraat 164 1061 AE, Amsterdam, the Netherlands; c Department of Pediatric Neurology, Emma Children's Hospital, Amsterdam UMC, Meibergdreef 9 1105, AZ, Amsterdam, the Netherlands

Zellweger Spectrum Disorders (ZSDs) are a heterogeneous group of autosomal recessive disorders characterized by a defect in peroxisome biogenesis and are caused by mutations in *PEX* genes. Because of the defect in peroxisome function, multiple metabolic pathways are impaired [ 1]. There is a phenotypic continuum ranging from mild to severe. Severe ZSDs are characterized by profound neonatal onset neurological symptoms, liver dysfunction, primary adrenal insufficiency, failure to thrive and craniofacial dysmorphism; these patients typically die during the first year of life [2,3].

We report a case of a neonate with a severe ZSD and hypoglycemia due to hyperinsulinism. Later, additional genetic testing revealed a second diagnosis of 16p11.2 deletion syndrome.

The male patient was the second child of non-consanguineous parents. Maternal history was unremarkable. Two brothers of father had died in infancy. The pregnancy was complicated by multiple fetal anomalies detected by prenatal ultrasound (including talipes equinovarus, facial dysmorphism and brain malformations). The parents did not want any further prenatal testing. There were no other complications of pregnancy and the patient was born at term with a birth weight of 3330 g (47th percentile).

After birth, clinical suspicion of severe ZSD was confirmed by a homozygous mutation (c.818insCTTG (p.Pro274Leufs*8)) in the *PEX6* gene detected by Whole Exome Sequencing (WES) targeting gene-panels ‘multiple congenital anomalies’ and ‘skeletal dysplasia’; and by abnormalities in the evaluation of biochemical biomarkers for peroxisomes (see Table 1). Both parents were carriers of the *PEX6* mutation. Medical records of the two brothers of father who had died in infancy showed the same homozygous *PEX6* mutation as the patient.

**Table 1:** Sample of relevant laboratory results and carbohydrate intake.

Day of life	9^a^	24^a^	25^a^	35^a^	40^a^	22–43
Result (reference range)						
Plasmalogens C16:0 in erytrocytes in % (5.5–8.1)	0.3					
Plasmalogens C18:0 in erythrocytes in % (11.8–19.4)	0.3					
C26:0 lysophosphatidylcholine in plasma in nmol/L (29–72)	167					
Dihydroxycholestanoic acid in plasma in μmol/L (0)	12.6					
Trihydroxycholestanoic acid in plasma in μmol/L (0–0.1)	37.6					
C29-dicarboxylic bile acid in plasma in μmol/L (0)	1.1					
Glucose (in blood) in mmol/L (>2.6)		2.2	2.4	1.9	1.9	1.7–7.7
Ketones (in blood) in mmol/L (<0.5)			0.3		<0.1	
Insulin in pmol/L (undetectable during hypoglycemia)		14		4		
C-peptide in nmol/L (0.3–1.2)		0.42				
Growth hormone in mIU/L		40.9				
Insulin-like growth factor in nmol/L (6–35)		10 (−1.4SD)				
Insulin-like growth factor-binding protein 3 in mg/L (1.3–4.5)		2.4				
Thyroid stimulating hormone in mIU/L (0.8–10)		3.6				
Free thyroxine in pmol/L (12–28)		20				
Adrenocorticotropic hormone in ng/L (<46)		20				
Aldosterone in pmol/L (62–980)		441				
Bilirubin (total) range in μmol/L (<17)						55–82
Bilirubin (conjugated) range in μmol/L (<5)						48–71
Alanine aminotransferase range in IU/L (<45)						69–115
Aspartate aminotransferase range in IU/L (<60)						153–236
Gamma-glutamyl Transpeptidase range in IU/L (<165)						76–145
Carbohydrate intake in mg/kg per minute		12.9	16.8	13.2		11.1–17.8

a Results from one blood sample.

The patient was hypotonic, required tube feeding and developed intractable epilepsy from day 6, which was treated with Levetiracetam. Magnetic resonance imaging of the brain showed polymicrogyria, pachygyria and periventricular cysts. Abdominal ultrasound showed bilateral renal cysts and dilated intrahepatic bile ducts. Liver function was normal at first, but later the patient developed cholestasis. Weight gain was stable at the 16th percentile.

The patient also had persistent recurrent hypoglycemic episodes, starting at birth (serum glucose 0.9 mmol/L). Insulin was measurable during hypoglycemia. An adrenal insufficiency was excluded by performing an adrenocorticotropic hormone (ACTH)-stimulation test.

The patient was treated with a high carbohydrate intake (11.1–17.8 mg/kg/min), but hypoglycemic episodes remained. See Table 1 for relevant laboratory results.

The patient was diagnosed with hyperinsulinism because of the following findings: measurable insulin during hypoglycemia, low blood ketones during hypoglycemia, the requirement of high carbohydrate intake to achieve euglycemia and hypoglycemic episodes during continuous tube feeding (see Table 1). Other causes of hypoglycemia in our patient were highly unlikely: adrenal insufficiency had been ruled out; there was no administration of blood glucose lowering medication; there was no malnutrition or sepsis and additional metabolic screening showed no signs of another inborn error of metabolism.

Later, additional genetic testing (Copy Number Variation (CNV)) revealed a de novo 16p11.2 deletion syndrome.

The patient died of respiratory insufficiency after 2 months.

To our knowledge only one study in 1972 described hypoglycemia in a patient with severe ZSD [4]. This reported patient presented profound episodes of deep hypoglycemia and died at 4 months of age. Autopsy revealed diffuse pancreatic islet cell hyperplasia.

In addition, hypoglycemia of unknown cause is described in the mild ZSD mouse model [5].

At first, the hyperinsulinemic hypoglycemia was thought to be another feature of ZSD. However, after receiving positive results of the 16p11.2 deletion, the hyperinsulinemic hypoglycemia was most likely caused by the 16p11.2 deletion syndrome, as it was described recently [6]. The exact causal mechanism of hyperinsulinism in 16p11.2 deletion is not yet known. Other features of 16p11.2 deletion syndrome are developmental delay, epilepsy, mild variable dysmorphism and increased chance of obesity [6].

In conclusion, we advise to consider the possibility of an additional genetic diagnosis when a symptom is not fitting with the initial diagnosis.
